# Model-Informed Precision Dosing during Infliximab Induction Therapy Reduces Variability in Exposure and Endoscopic Improvement between Patients with Ulcerative Colitis

**DOI:** 10.3390/pharmaceutics13101623

**Published:** 2021-10-06

**Authors:** Ruben Faelens, Zhigang Wang, Thomas Bouillon, Paul Declerck, Marc Ferrante, Séverine Vermeire, Erwin Dreesen

**Affiliations:** 1 Department of Pharmaceutical and Pharmacological Sciences, Katholieke Universiteit Leuven, 3000 Leuven, Belgium; ruben.faelens@kuleuven.be (R.F.); zhigang.wang@kuleuven.be (Z.W.); thomas.bouillon@bionotus.com (T.B.); paul.declerck@kuleuven.be (P.D.); 2 Department of Gastroenterology and Hepatology, University Hospitals Leuven, 3000 Leuven, Belgium; marc.ferrante@uzleuven.be (M.F.); severine.vermeire@uzleuven.be (S.V.); 3 Department of Chronic Diseases and Metabolism, Katholieke Universiteit Leuven, 3000 Leuven, Belgium

**Keywords:** infliximab, monoclonal antibody, ulcerative colitis, inflammatory bowel disease, endoscopy, population pharmacokinetics-pharmacodynamics, simulations, therapeutic drug monitoring, model-informed precision dosing

## Abstract

Model-informed precision dosing (MIPD) may be a solution to therapeutic failure of infliximab for patients with ulcerative colitis (UC), as underexposure could be avoided, and the probability of endoscopic improvement (pEI; Mayo endoscopic subscore ≤ 1) could be optimized. To investigate in silico whether this claim has merit, four induction dosing regimens were simulated: 5 mg/kg (label dosing), 10 mg/kg, covariate-based MIPD (fat-free mass, corticosteroid use, and presence of extensive colitis at baseline), and concentration-based MIPD (based on the trough concentration at day 14). Covariate- and concentration-based MIPD were chosen to target the same median area under the infliximab concentration-time curve up to endoscopy at day 84 (AUC_d84_), as was predicted from 10 mg/kg dosing. Dosing at 5 mg/kg resulted in a mean ± standard deviation pEI of 55.7 ± 9.0%. Increasing the dose to 10 mg/kg was predicted to improve pEI to 65.1 ± 6.1%. Covariate-based MIPD reduced variability in exposure and pEI (65.1 ± 5.5%). Concentration-based MIPD decreased variability further (66.0 ± 3.9%) but did so at an increased average dose of 2293 mg per patient, as compared to 2168 mg for 10 mg/kg dosing. Mean pEI remained unchanged between 10 mg/kg dosing and MIPD, since the same median AUC_d84_ was targeted. In conclusion, quantitative simulations predict MIPD will reduce variability in exposure and pEI between patients with UC during infliximab induction therapy.

## 1. Introduction

Infliximab is a monoclonal antibody that binds and neutralizes the functional activity of tumor necrosis factor-alpha (TNFα). Based on the results of the landmark Active Ulcerative Colitis Trials (ACT) 1 and 2, infliximab was approved for inducing and maintaining remission in patients with moderate-to-severe ulcerative colitis (UC) [1]. In these studies, endoscopic improvement (defined as a Mayo endoscopic subscore ≤ 1) was achieved in about 60% of patients after administration of three infliximab infusions (5 mg/kg body weight, at weeks 0, 2, and 6; endoscopy at week 8). In post-marketing studies, endoscopic improvement rates were lower (e.g., 47% in Brandse et al. [2]), making unpredictable outcomes of infliximab induction therapy a challenge [2,3,4,5].

Dose finding in ACT 1 and 2 failed to show a consistent benefit of 10 mg/kg dosing over 5 mg/kg dosing [1]. However, higher infliximab serum concentrations during induction therapy were found to correlate with short-term endoscopic improvement, as well as long-term relapse-free and colectomy-free survival [6]. To date, the infliximab exposure-response relationship in patients with UC has been well-established [4,7,8,9]. Consequently, it has been hypothesized that targeting infliximab to a predefined “optimal” exposure has the potential to improve the response rate and identify primary non-responders (defined as non-response despite optimal infliximab exposure) [2,10,11]. To date, most therapeutic drug monitoring (TDM) studies of infliximab focus on maintenance therapy, whereas induction therapy is relatively unexplored. Moreover, the utility of TDM of infliximab in patients with UC remains controversial because of poor evidence from prospective TDM studies [10,12,13,14]. One potential reason for the weak evidence can be the use of inefficient TDM algorithms (analogous flowcharts and decision trees) in these TDM studies [15]. Therefore, model-informed precision dosing (MIPD), a more efficient and precise dose optimization strategy as compared to analogous TDM, has been suggested as a way out of this dilemma [15,16].

MIPD can be implemented through either a priori or a posteriori dose optimization, both utilizing a population pharmacokinetic (popPK) model that serves as a prior. A priori dose optimization is done by involving patient’s covariates/characteristics that explain between- and within-subject variability, while a posteriori dose optimization (Bayesian forecasting) is based on previous infliximab serum concentration measurements [17]. Through these two approaches, the MIPD software tool can recommend a dose that facilitates attainment of the therapeutic target exposure. Patient covariates such as C-reactive protein (CRP), serum albumin, antibodies to infliximab (ATI), body weight or fat-free mass, and fecal calprotectin have previously been identified in popPK modeling studies [9,18].

In a previous popPK and exposure-response modeling analysis, we identified the relation between the area under the infliximab concentration-time curve up to endoscopy at day 84 (AUC_d84_) and the probability of endoscopic improvement at day 84 [18]. Based on these results, we suggested that increased exposures would result in better clinical outcomes. We further suggested that any increased drug consumption may be offset through the use of MIPD. In the present work, we investigated these claims further by performing population simulations of these different dosing scenarios and comparing exposures, probability of endoscopic improvement, and average drug consumption.

## 2. Materials and Methods

### 2.1. Population Pharmacokinetic and Exposure-Response Models

A previously published one-compartment popPK model with interindividual and interoccasion variability was used to simulate infliximab exposure [18]. This model was built on a total of 583 samples from 204 patients with UC, and included C-reactive protein (CRP), serum albumin, and fat-free mass (FFM) as time-varying covariates, and Mayo endoscopic subscore, presence of extensive colitis, and corticosteroid use as baseline covariates.

Even though dose proportionality applies, when administering a higher dose of infliximab (*cf.* Section 2.3*. Dosing Scenarios*), a more positive disease evolution is expected, thereby influencing the time-course of CRP and serum albumin, both acute phase proteins, and possibly fat-free mass as well. Since the original dataset used for popPK model building did not include patients on higher infliximab doses (*cf.* Section 2.2 *Virtual Population*), and to avoid bias in the scenarios with higher dosing, we chose to re-estimate the model without these covariates. In theory, this should increase the unexplained interoccasion variability and residual error instead.

The logistic regression exposure–response model was adapted as well. The model was built on a subset of 159 patients and fitted the original data well [18]. However, this model predicted an ever-increasing probability of endoscopic improvement with increasing infliximab exposure. This could not be reconciled with the current line of thinking for infliximab treatment in UC, which assumes the existence of intrinsic non-responders [19]. The model was adapted to introduce maximum transition probabilities E_max,3__→2_ and E_max,2__→1/0_ for transitioning from a severe disease state (Mayo endoscopic subscore 3) to a moderate disease state (Mayo endoscopic subscore 2) and from a moderate disease state to endoscopic improvement (Mayo endoscopic subscore 1 or 0), respectively. Likelihood profiling was performed to identify the confidence bound for these parameters [20]. These E_max_ parameters were varied across a wide range of values and the associated AUC_50_s (i.e., the infliximab exposures required to achieve half-maximal transition probabilities) were estimated, yielding a log-likelihood (LL) estimate for each parameter set. Estimates with Δ2LL = 3.84 showed the lower 95% confidence bound for the exposure–response model. These parameter estimates were then used for subsequent simulations.

### 2.2. Virtual Population

To construct the virtual population for the dosing simulations, the original clinical dataset was used [7]. Only patients with a baseline Mayo endoscopic subscore of 2 or 3 were included, resulting in a source dataset of 194 patients. This dataset was expanded through Monte Carlo sampling of interindividual variability (200 samples per individual patient), yielding a total of 38,800 virtual patients.

Baseline covariates were collected in a study conducted in accordance with the principles of good clinical practice and the Declaration of Helsinki. All patients provided written informed consent prior to participation in the Ethics Committee-approved IBD Biobank [B322201213950/S53684], whereby patients’ characteristics and samples were collected prospectively on a series of predefined time points.

### 2.3. Dosing Scenarios

Four distinct dosing scenarios were evaluated. First, a standard dosing regimen of 5 mg/kg at days 0, 14, and 42 was applied to all virtual patients. Based on the exposure–response analysis of the original dataset, there was support for a higher dose [18]. Therefore, 10 mg/kg was evaluated as a second dosing scenario.

We aimed for covariate-based and concentration-based MIPD to result in the same mean predicted probability of endoscopic improvement as in the 10 mg/kg dosing scenario. Therefore, MIPD scenarios were designed to target the same median AUC_d84_ as was predicted from the 10 mg/kg dosing scenario. The third dosing scenario was purely based on the covariates (a priori MIPD). The popPK model was used to determine the covariate-based dose required to hit the exposure target associated with the predefined probability of endoscopic improvement.

Finally, Bayesian forecasting (a posteriori MIPD) was evaluated as a fourth dosing scenario. The first dose was the same as in the covariate-based MIPD scenario. The sampled interindividual variability was used to simulate the trough concentration on day 14 resulting from the covariate-based first dose. Residual error was sampled and added to this concentration. This simulated concentration was subsequently used to perform an empirical Bayesian estimation of the patient’s individual PK parameters. These individual estimates were then used to adapt the subsequent doses at days 14 and 42. Both doses were adapted to the same value, predicted to result in an AUC_d84_ exposure metric resulting in the target probability of endoscopic improvement.

### 2.4. Evaluation of Dosing Scenarios

The mean dose per patient and resulting exposures (AUC_d84_) in each scenario were evaluated graphically as density plots. To quantify efficacy, the mean probability of endoscopic improvement was evaluated, as this reflects the expected fraction of patients attaining endoscopic improvement. Additionally, the mean overall dose per patient was evaluated. Finally, a robustness analysis was performed to determine whether our conclusions hold for other E_max_ parameter values.

### 2.5. Software

The adapted popPK and exposure–response models were estimated using NONMEM (version 7.4.3; Icon Development Solutions, Gaithersburg, MD, USA). Simulation of the dosing scenarios was performed using R (version 4.0.2; R Foundation for Statistical Computing, R Core Team, Vienna, Austria) with RxODE [21] and *tdmore*. The *tdmore* R package was developed at KU Leuven to perform simulation and evaluation of MIPD. It is available as open-source at github.com/tdmore-dev/tdmore (accessed on 27 August 2021). The NONMEM code and *tdmore* R code are provided in the Supplementary File.

## 3. Results

### 3.1. Population Pharmacokinetic and Exposure-Response Models

The popPK model was adapted to include only covariates at baseline. As expected, the interindividual variability on the elimination rate constant and the proportional residual error increased (Table S1, Supplementary Material). A visual predictive check of the updated popPK model is available in Figure S1.

**Figure 1:**
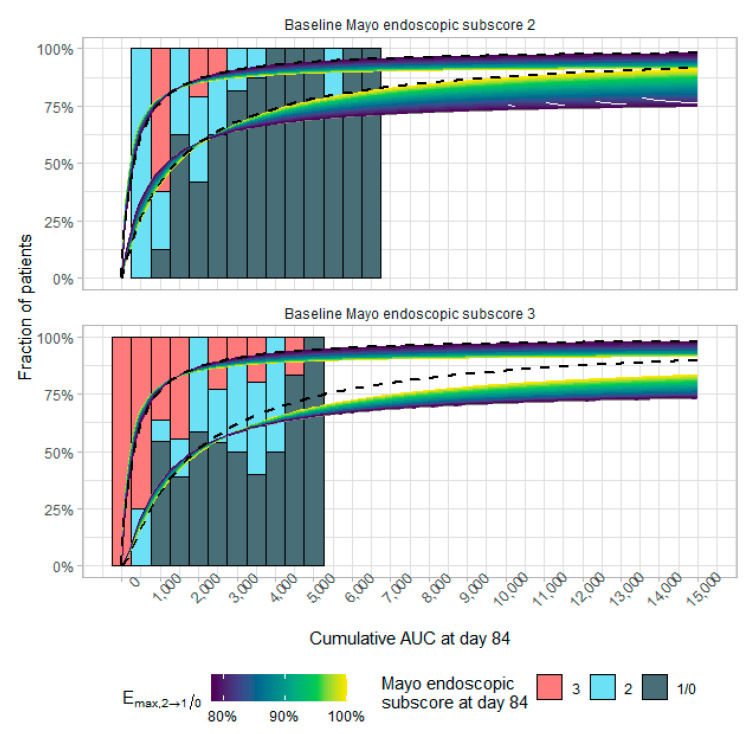
Exposure–response dataset (bars), binned per cumulative area under the curve (AUC) at day 84 and categorized according to Mayo endoscopic subscore at day 84, and corresponding simulated exposure–response models (lines representing the fraction of patients achieving a Mayo endoscopic subscore ≤1 [lower line] and ≤2 [upper line]). The original exposure–response model of Dreesen et al. [18] is shown as a black dashed line (E_max,3__→2_ and E_max,2__→0/1_ are both 100%). Colored lines represent models at Δ2LL = 3.84 with E_max,3__→2_ 93% and different E_max,2__→1/0_ values. All presented models fit the exposure–response dataset equally well (at α = 0.05) but have different predictions outside the observed exposure range.

Likelihood profiling of the exposure–response model showed a wide range of probable E_max,3__→2_–E_max,2__→1/0_ pairs. In Figure S2, the likelihood profile is shown for different parameter combinations. The Δ2LL = 3.84 contour line in red shows parameter combinations limits for E_max,3__→2_–E_max,2__→1/0_ of either 92.6–100% or 100–78.4%. Figure 1 shows the simulated PD model at parameter estimates with associated Δ2LL = 3.84. Based on this plot, E_max,2__→1/0_ = 78.4%/E_max,3__→2_ = 100% was selected for further simulations, as the most “pessimistic” scenario. The remainder of the possible parameter values were explored in the sensitivity analysis.

### 3.2. Dosing Simulations: Exposure and Efficacy

**Figure 2:**
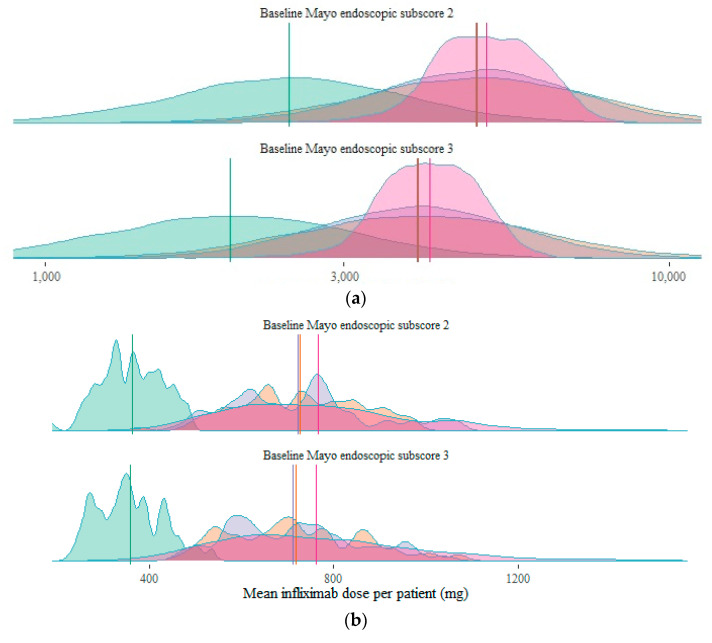

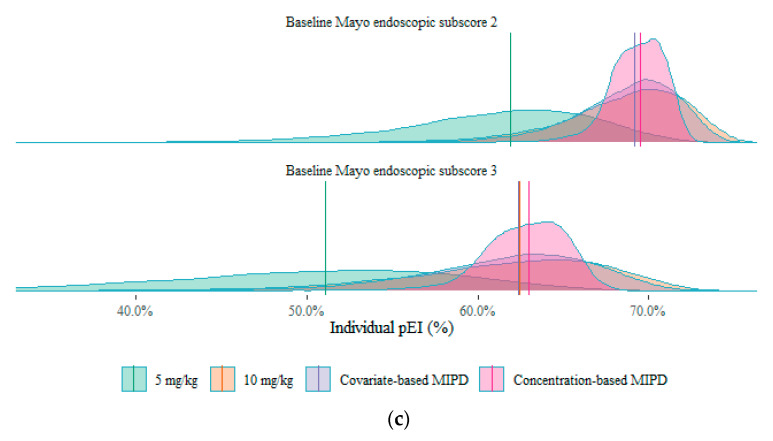
(**a**) Density plots of exposure in each of the four dosing scenarios, per baseline Mayo endoscopic subscore. Vertical lines show median exposure per scenario. (**b**) Density plots of the mean doses in each of the four dosing scenarios, per baseline Mayo endoscopic subscore. Vertical lines show overall mean dose per scenario. (**c**) Density plots of the individual probability of endoscopic improvement in each of the four dosing scenarios, per baseline Mayo endoscopic subscore. Vertical lines show overall mean pEI per scenario. CAUC, cumulative area under the curve; MIPD, model-informed precision dosing; pEI, probability of endoscopic improvement.

**Figure 3:**
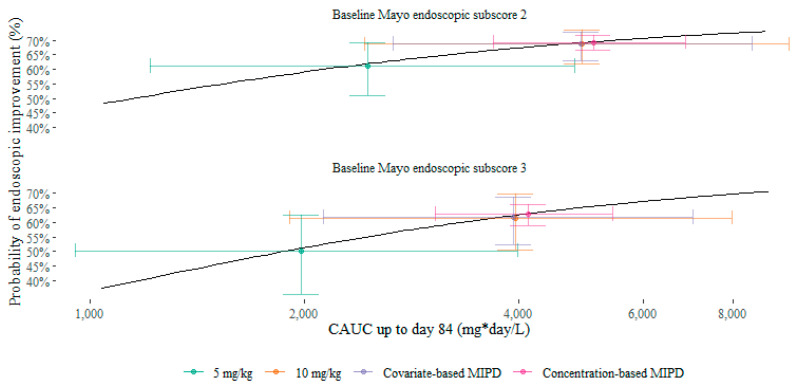
Predicted mean probability of endoscopic improvement versus median exposure, with 90% prediction interval in each scenario, per baseline Mayo endoscopic subscore. Black lines show model-predicted response. CAUC, cumulative area under the curve; MIPD, model-informed precision dosing.

Simulation results are summarized in Table 1 and will be presented hereafter as median [95% prediction interval] for exposure (AUC_d84_) and mean ± standard deviation for probability of endoscopic improvement. As exposure and efficacy targets differ depending on the baseline endoscopic disease severity, results are reported for baseline Mayo endoscopic subscores of 2 (moderate disease severity; reported first) and 3 (high disease severity; reported second), separately. Exposures and associated probabilities of endoscopic improvement are shown in Figure 2 and Figure 3.

The 5 mg/kg dosing scenario resulted in an AUC_d84_ of 2455 [1215–4805] mg × day/L and 1979 [953–3990] mg × day/L, for baseline Mayo endoscopic subscore 2 and 3, respectively. This resulted in a predicted probability of endoscopic improvement of 61.2 ± 5.5% and 50.3 ± 8.4%. By increasing the dose to 10 mg/kg, exposure doubled to 4910 [2431–9609] mg × day/L and 3958 [1906–7981] mg × day/L. Probabilities of endoscopic improvement also increased to 68.6 ± 3.6% and 61.6 ± 6.1%.

Adapting the dose based on relevant covariates allowed more precise dosing, as between-population-variability can be taken into account. As can be seen in Figure 2, covariate-based MIPD resulted in the same median exposure as 10 mg/kg dosing, at a reduced variability (4895 [2661–8522] mg × day/L and 3933 [2123–7045] mg × day/L, for baseline Mayo endoscopic subscore 2 and 3, respectively). Dose adaptation based on the trough concentration measured at day 14 (Bayesian forecasting) further reduced this variability (5095 [3683–6879] mg × day/L and 4125 [3056–5431] mg × day/L). The probability of endoscopic improvement followed a similar pattern, with similar mean probabilities across 10 mg/kg dosing, covariate-based MIPD, and concentration-based MIPD.

### 3.3. Dosing Simulations: Drug Consumption

Looking at the average infliximab dose used per patient (see also Figure 2), 5 mg/kg dosing resulted in 1084 mg per patient, and 10 mg/kg dosing doubled the dose usage to 2168 mg per patient. Covariate-based MIPD used an average of 2151 mg per patient. Concentration-based MIPD used, at average, 2293 mg per patient.

### 3.4. Sensitivity Analysis

The analysis presented above assumed an E_max_ plateau of 78% for the probability of transitioning from a Mayo endoscopic subscore of 2 (moderate disease severity) to a Mayo endoscopic subscore of 0 or 1 (endoscopic improvement). This plateau benefited MIPD, as overexposed patients were dose-reduced without significantly reducing the probability of endoscopic improvement, while underexposed patients were dose-increased, thereby significantly increasing the probability of endoscopic improvement.

Repeating our simulation study with higher values for E_max,2__→1/0_ decreased this benefit, further favoring 10 mg/kg dosing, as is illustrated in Figure 2. Other parameter combinations at Δ2LL = 3.84, as well as for the base model (Δ2LL = 0), consistently showed less favorable results for MIPD.

At E_max,3__→2_–E_max,2__→1/0_ of 92.6–100%, the probability of endoscopic improvement for 10 mg/kg dosing was 77.0% [62.7–86.5%] and 65% [48.1–76.7%], for baseline Mayo endoscopic subscore 2 and 3, respectively, at an average drug consumption of 2168 mg per patient. Bayesian forecasting resulted in a probability of endoscopic improvement of 77.6% [71.7–82.3%] and 65.8% [59.4–70.9%], for baseline Mayo endoscopic subscore 2 and 3, at an average drug consumption of 2293 mg per patient.

## 4. Discussion

In this study, we compared four possible dosing scenarios for infliximab induction therapy in patients with UC: 5 mg/kg weight-based dosing (label dosing), and three dosing strategies with increased exposure: 10 mg/kg weight-based dosing, covariate-based MIPD, and concentration-based MIPD, all with unchanged timing of the infusions (day 0, 14, and 42). The 10 mg/kg dosing scenario was predicted to significantly improve endoscopic outcomes as compared to 5 mg/kg dosing. By design, MIPD (based on either covariates or the day 14 trough concentration) resulted in the same median exposure (AUC_d84_) and, consequently, the same mean probability of endoscopic improvement as observed in the 10 mg/kg dosing scenario. MIPD was predicted to successfully adapt individual patient doses, reducing the interindividual variability in infliximab exposure and, with it, the probability of endoscopic improvement, thereby providing “more equal” chances of endoscopic remission to all patients. Surprisingly, it did so at a higher average drug consumption per patient. Underexposed patients indeed received a relative dose increase, while overexposed patients received a relative dose decrease. However, this is a non-zero sum, as, e.g., 10 mg × 2 + 10 mg × 0.5 > 10 mg + 10 mg. Therefore, our simulation study showed improved outcomes under 10 mg/kg dosing as compared to 5 mg/kg dosing and showed additional benefit of MIPD over 10 mg/kg for reducing variability in exposure and efficacy between patients; however, at a higher direct drug cost. Performing MIPD may thus require a willingness to “pay for equality” amongst patients [22]. Consequently, we may consider shifting focus from outcome rates at the population level (the traditional industry perspective) to outcome chances at the individual patient level. It is at the individual patient level that MIPD may show value. Since the majority of patients attain the target under empirical dosing, it is important that future MIPD studies are restricted to vulnerable populations, such as patients with acute severe ulcerative colitis [23].

The simulations described in this work were based on a previously established popPK model and exposure–response model of infliximab [18]. These models described the relation between the infliximab dose and exposure, and the AUC_d84_ and the probability of endoscopic improvement at day 84, respectively. The models were established on a dataset of 204 patients with moderate-to-severe UC. Since the majority of the infliximab doses in the original cohort were 5 mg/kg (approximately 90%), and only about 10% of the doses were 10 mg/kg, it should be noted that the exposure–response model was built on a relatively limited range of exposures. The exposures simulated in the present work exceed this range. However, this weakness was mitigated by a thorough analysis of exposure–response model parameter confidence intervals and likelihood profiling, and a sensitivity analysis. Our findings hold throughout the full range of probable model parameter values.

The exposure–response model assumes a causal effect between exposure and response. Previous clinical studies have indeed found a correlation between low trough concentrations and primary nonresponse to anti-TNFα therapy [24]. However, the causality assumed in our exposure–response model was never established in clinical studies. In light of this, time-varying disease-related covariates may instead be simulated in a joint model, avoiding potential underestimation of exposure at higher doses and reduced disease severity. Further research is needed to definitively establish whether non-response at low trough concentrations is due to mechanistic failure (pharmacodynamic [PD] failure) or underexposure (PK failure), as others have attempted to model this distinction [25,26]. Underexposure can be resolved through dose increase, while the mechanistic failure suggests switching to a different drug with another mechanism of action. A more fine-grained model of continuous endpoints may distinguish between PK and PD failure.

High exposure to infliximab may pose safety concerns. The 10 mg/kg dosing may result in very high exposures, which were predicted in the present study to be beneficial to patients. In reality, these highly exposed patients may present with adverse drug reactions such as infections, especially in the elderly, and MIPD may benefit these patients by reducing toxicity [27,28,29].

Our findings seemingly contradict the pivotal ACT 1 and 2 trials [1], which showed no significant difference between 5 mg/kg and 10 mg/kg in endoscopic improvement rates on day 56 of therapy. Nevertheless, in the post-hoc PK-PD analysis of ACT 1 and 2, the exposure–response relationship has been established [6]. It would be worthwhile to repeat the presented modeling and simulation exercise, including the data from these pivotal trials. Notwithstanding these results, clinical trials are currently underway, evaluating an intensified induction regimen of 10 mg/kg [30].

MIPD of infliximab has been implemented in clinical practice mainly in tertiary care centers, however, even there, the confidence in MIPD is crumbling as the results of the landmark TAXIT, TAILORIX, and NOR-DRUM trials do not live up to expectations [12,14,31]. Our research showed that in silico simulations are a low-cost alternative to these clinical studies. Nevertheless, the translation of findings from a virtual trial into the real world may be challenged by noise due to, for example, sampling and measurement errors, rounding of doses and dosing intervals, etc. [32].

MIPD is classically used to improve the probability of target attainment, with the target window defined by efficacy and toxicity. In this context, efficacy is ever-increasing with higher exposures, while a dose increase is largely limited by cost rather than toxicity. It may be interesting to quantify the effect of infliximab as quality-adjusted life years (QALY) instead, allowing a direct comparison to increased cost and a straightforward optimization of QALY/cost.

In summary, we performed simulations to illustrate and predict the impact of three dosing strategies for increasing infliximab exposure during induction therapy as compared to 5 mg/kg weight-based label dosing, thereby improving the probability of endoscopic improvement. The use of 10 mg/kg dosing was indeed predicted to improve the probability of endoscopic improvement to 65.1% at an average drug consumption of 2168 mg per patient during induction therapy. Individualized dose adaptation could maintain the same mean probability of endoscopic improvement while reducing variability between individual patients. Although MIPD showed benefit for reducing variability in exposure and efficacy between patients, this comes at a higher direct drug cost as compared to 10 mg/kg weight-based dosing.

## Figures and Tables

**Table 1:** Summary of the simulation results.

		AUC_d84_ (mg/L × Day)		pEI (%)		Cumulative Dose (mg)	
Baseline Mayo Endoscopic Subscore	Dosing scenario	median	[90%PI]	mean	±sd	mean	±sd
2	5 mg/kg	2455	[1215–4805]	61.2	±5.51	1090	±196
	10 mg/kg	4910	[2431–9609]	68.6	±3.60	2181	±393
	Covariate-based MIPD	4895	[2661–8522]	68.7	±3.08	2166	±443
	Concentration-based MIPD	5095	[3683–6879]	69.3	±1.67	2298	±613
3	5 mg/kg	1979	[953–3990]	50.3	±8.36	1078	±214
	10 mg/kg	3958	[1906–7981]	61.6	±6.05	2155	±428
	Covariate-based MIPD	3933	[2123–7045]	61.7	±5.06	2137	±417
	Concentration-based MIPD	4125	[3056–5431]	62.8	±2.51	2287	±643
Combined (2:3; 49%:51%)	5 mg/kg	2210	[1049–4448]	55.7	±8.96	1084	±205
	10 mg/kg	4419	[2098–8895]	65.1	±6.11	2168	±411
	Covariate-based MIPD	4372	[2302–7940]	65.1	±5.46	2151	±431
	Concentration-based MIPD	4561	[3209–6516]	66.0	±3.91	2293	±628

The systematically lower exposure at a baseline Mayo endoscopic subscore of 3 (severely active ulcerative colitis), as compared to a baseline Mayo endoscopic subscore of 2 (moderately active ulcerative colitis), may mechanistically be explained by a higher target load (target-mediated drug disposition) and protein-losing enteropathy (fecal drug loss). AUC_d84_, the area under the infliximab concentration-time curve from baseline up to endoscopy at day 84 (week 12); MIPD, model-informed precision dosing; pEI, probability of endoscopic improvement; PI, prediction interval; q, quantile; sd, standard deviation.

## Data Availability

The data presented in this study are available in the research article and supplementary material here.

## Supplementary Materials

The following are available online at https://www.mdpi.com/article/10.3390/pharmaceutics13101623/s1, Figure S1: Prediction-corrected visual predictive check of the original population pharmacokinetic model, Figure S2. Log-likelihood surface for the exposure-response model, Table S1. Parameter estimates of the original and adapted population pharmacokinetic models.
