# Supplementary material for: Model-Informed Precision Dosing during Infliximab Induction Therapy Reduces Variability in Exposure and Endoscopic Improvement between Patients with Ulcerative Colitis
Source: Pharmaceutics. 2021 Oct 6;13(10):1623. doi: 10.3390/pharmaceutics13101623 (PMC8537637; doi:10.3390/pharmaceutics13101623)
Supplement: Supplementary file 1 [file pharmaceutics-13-01623-s001.zip › pharmaceutics-1377416-supplementary.pdf]

pharmaceutics

MDPI

Supplementary Materials: Model-Informed Precision Dosing during Infliximab Induction Therapy Reduces Variability in Exposure and Endoscopic Improvement between Patients with Ulcerative Colitis

Ruben Faelens, Zhigang Wang, Thomas Bouillon, Paul Declerck, Marc Ferrante, Séverine Vermeire and Erwin Dreesen

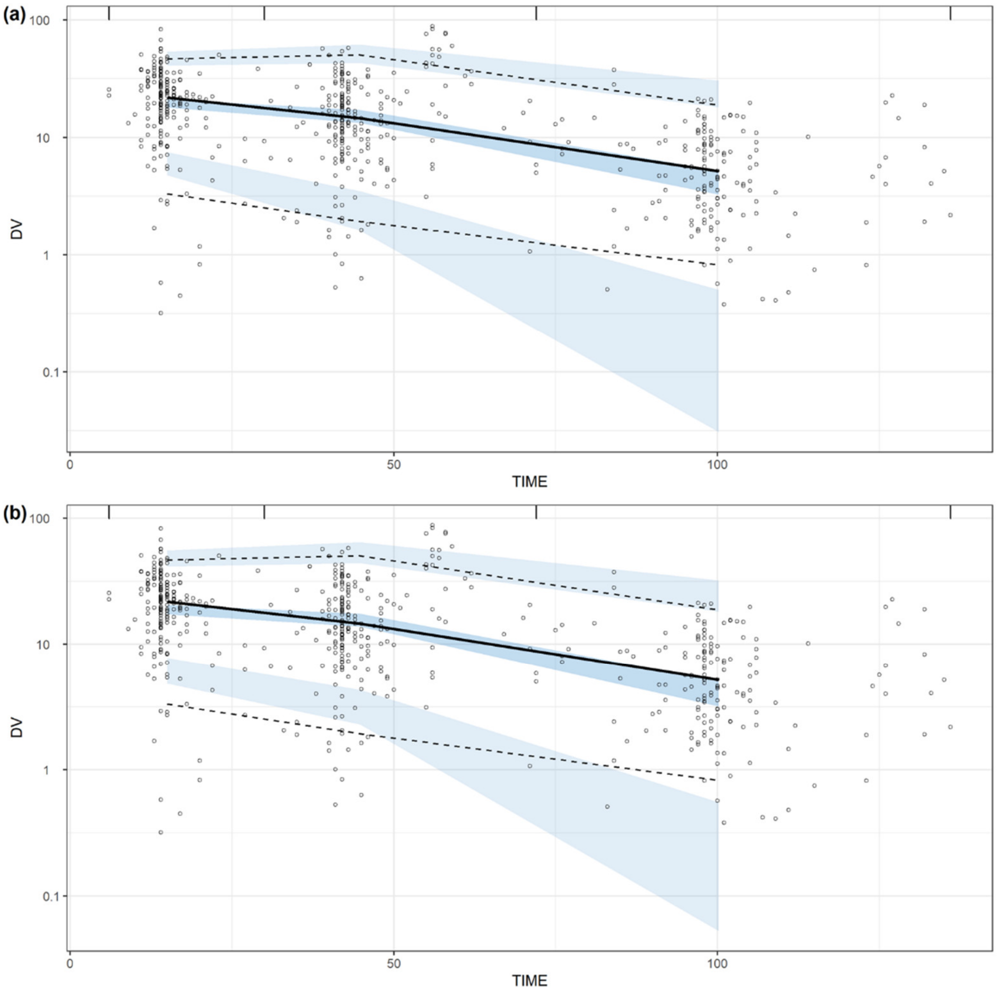

Figure S1. Prediction-corrected visual predictive check of the original population pharmacokinetic model **(a)** and the adapted population pharmacokinetic model **(b)**. The solid line connects the observed median prediction-corrected infliximab serum concentrations (mg/L) per bin. The dashed lines connect the 5th and 95th percentiles of the prediction-corrected observations. Shaded areas indicate the 95% confidence interval of the median and 5th and 95th percentiles of the simulated values. The observed prediction-corrected infliximab concentrations are represented by black open circles.

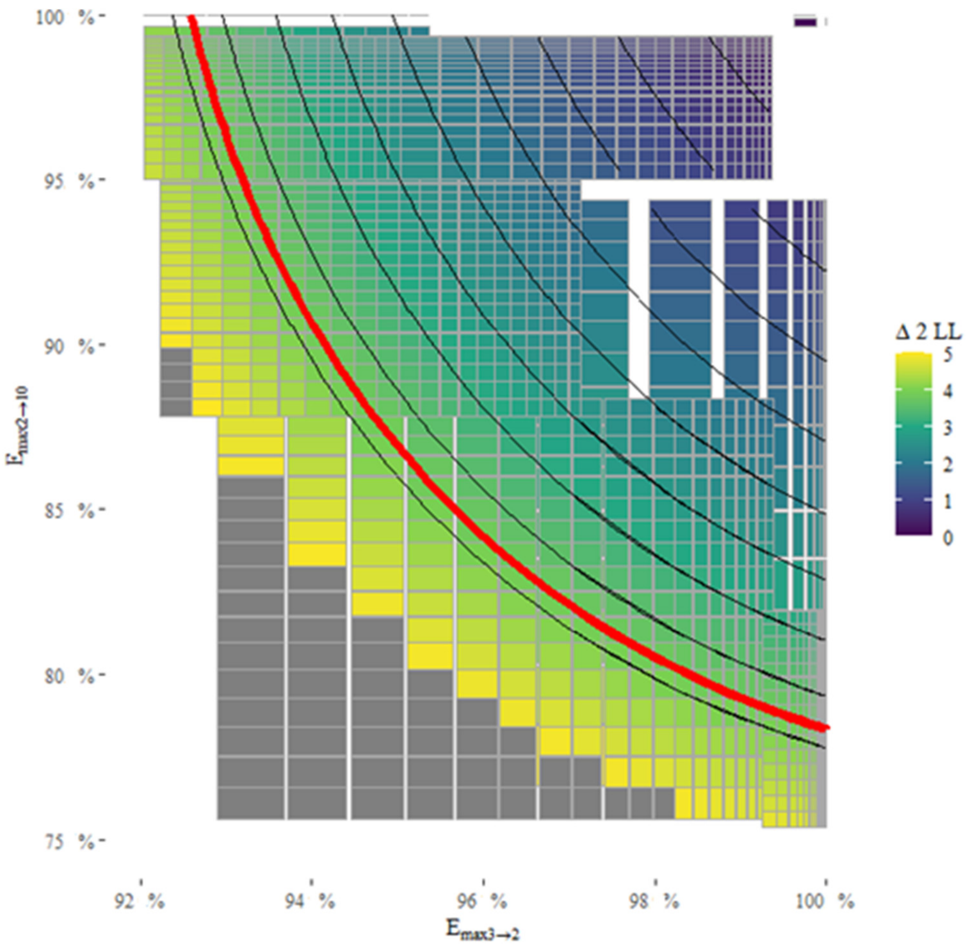

Figure S2. Log-likelihood surface for the exposure-response model. The maximum transition probabilities $E_{max,2\rightarrow3}$ and $E_{max,2\rightarrow1/0}$ were fixed while associated AUC_{50} values (infliximab exposure metrics associated with a half-maximum transition probability) were estimated. The top right point at 100%–100% represents the original exposure-response model as reported by Dreesen *et al*. [18]. All models top right of the red contour line has a Δ2LL <3.84.

Table S1. Parameter estimates of the original and adapted population pharmacokinetic models.

**Parameter**	**Original Model [18]** *Estimate*	**(OFV=2696)** *(RSE) (%)*	**Adapted Model** *Estimate*	**(OFV=2784)** *RSE (%)*
**Typical values**				
Elimination rate constant (/d)				
baseline Mayo endoscopic subscore 1 (/d)	0.0521	(11)	0.0422	(14)
baseline Mayo endoscopic subscore 2 (/d)	0.0543	(3.6)	0.0463	(3.8)
baseline Mayo endoscopic subscore 3 (/d)	0.0667	(8.1)	0.0570	(5.0)
albumin	-0.808	(39)		
C-reactive protein	0.0859	(25)		
Volume of distribution (L)	6.34	(3.2)	6.97	(5.0)
fat-free mass	0.544	(29)	0.517	(39)
Corticosteroids [1]	1.33	(6.9)	1.30	(7.7)
Extensive colitis [1]	1.23	(0.019)	1.25	(6.9)
**Interindividual variability** [2]				
Elimination rate constant (CV%)	29.8	(7.8)	33.4	(5.7)
Volume of distribution (CV%)	26.5	(20)	23.6	(41)
**Interoccasion variability**				

Parameter	Original Model [18] *Estimate*	(OFV=2696) *(RSE) (%)*	Adapted Model *Estimate*	(OFV=2784) *RSE (%)*
Elimination rate constant (CV%)	18.7	(17)	6.70	(100)
**Residual variability**				
Proportional residual error (CV%)	19.2	(9.4)	32.9	(5.8)
Additive residual error (mg/L)	0.300	FIX	0.300	FIX

[1] The corticosteroid and extensive colitis effects were modelled as a fold change compared with the reference of no corticosteroids and no extensive colitis, respectively.
[2] Inter-individual CV% was calculated as sqrt(exp(omega)-1). CV, coefficient of variation; OFV, objective function value; RSE: relative standard error.

NONMEM code of the adapted population pharmacokinetic model

```
$PROBLEM     PopPK analysis of infliximab Induction therapy

$INPUT       ID TIME DV MDV EVID AMT RATE OCC EXTCOL MPRE CRP ALB CS WT HT SEX
             ; EXTCOL: Extensive colitis
             ; MPRE:   Mayo endoscopic score pre-induction
             ; CRP:    C-reactive protein
             ; ALB:    Albumin
             ; CS:     Corticosteroids
             ; WT:     Body weight
             ; HT:     Body height
             ; SEX:    Sex (female 0, male 1)

$DATA        PKCOV.csv IGNORE=@ IGNORE(DV .EQ. 0.15) ; exclude the BLQ data

$SUBROUTINE ADVAN6 TRANS1 TOL=6

$MODEL       COMP=(CENTRAL,DEFDOS,DEFOBS) COMP=(CUMAUC)

$PK BMI=WT/(HT**2)
    IF (SEX.EQ.1) THEN
                        FFM= (9.27*1000*WT)/((6.68*1000)+(216*BMI)) ;male
                  ELSE
                        FFM= (9.27*1000*WT)/((8.78*1000)+(244*BMI)) ;female
                  ENDIF

FLAG1=0
FLAG2=0
FLAG3=0
FLAG4=0

IF (OCC .EQ. 1) FLAG1=1
IF (OCC .EQ. 2) FLAG2=1
IF (OCC .EQ. 3) FLAG3=1
IF (OCC .EQ. 4) FLAG4=1

MPRE1=0
MPRE2=0
MPRE3=0
MPRE4=0

IF (MPRE.EQ.-99) MPRE1=1
IF (MPRE.EQ.1)   MPRE2=1
IF (MPRE.EQ.2)   MPRE3=1
IF (MPRE.EQ.3)   MPRE4=1

TVK = (MPRE1*THETA(4) + MPRE2*THETA(3) + MPRE3*THETA(2) + MPRE4*THETA(1))
TVV = THETA(6) *(THETA(5)**CS) *((FFM/52)**THETA(7)) *(THETA(8)**EXTCOL)

K   = TVK * EXP(ETA(1) +FLAG1*ETA(3)+FLAG2*ETA(4)+FLAG3*ETA(5)+FLAG4*ETA(6))
V   = TVV * EXP(ETA(2))

CL = V*K
S1 = V

$DES DADT(1) =  -K*A(1)
     DADT(2) =  A(1)/S1
     CAUC = A(2) ; cumulative AUC

$ERROR IPRED = F
       IRES  = DV-IPRED
       Y     = IPRED*(1+ERR(1))+ERR(2)
       IWRES = IRES/(SQRT(IPRED**2*SIGMA(1,1)+SIGMA(2,2)))

$THETA (0, 0.057)  ; KE MPRE3
       (0, 0.0463) ; KE MPRE2
       (0, 0.0422) ; KE MPRE1
```

```
       (0, 0.617)  ; KE MPRE-99
       (1.3)       ; CS on V
       (6.97)      ; TVV
       (0.517)     ; FFM on V
       (1.25)      ; EXTCOL on V

$OMEGA 0.106.      ;ETA(1)
       0.5         ;ETA(2)
$OMEGA BLOCK(1) 0.054 ;IOV
$OMEGA BLOCK(1) SAME
$OMEGA BLOCK(1) SAME
$OMEGA BLOCK(1) SAME

$SIGMA 0.103
       0.09 FIX

$ESTIMATION MAXEVAL=9999 NOABORT PRINT=1 NSIG=2 METHOD=1 INTER LAPLACE
$COVARIANCE

$TABLE ID DV TIME MDV EVID PRED IPRED RES WRES CWRES IWRES K CL V ONEHEADER NOPRINT NOAPPEND
FILE=PKCOV.sdtab
$TABLE ID K CL V ETA(1) ETA(2) ETA(3) ETA(4) ETA(5) ETA(6) FIRSTONLY NOAPPEND NOPRINT
FILE=PKCOV.patab
$TABLE ID EXTCOL MPRE CS SEX ONEHEADER NOPRINT FILE=PKCOV.catab
$TABLE ID CRP ALB WT HT ONEHEADER NOPRINT FILE=PKCOV.cotab
```

NONMEM code of the adapted exposure-response model

```
$PROBLEM MPRE==2,3, CAUC drives change

$INPUT ID DV C14 CAUC14 MPRE MPST CDOS CAUC TIPD
       ; TIPD: Time to PD assessment (post-induction endoscopy)

$DATA PKPD.csv IGNORE=@ IGNORE(MPRE.EQ.1) IGNORE(TIPD.LE.30) IGNORE(TIPD.GE.132)

$PRED PKMETRIC = CAUC
      EMAX1=1/(1+exp(-THETA(6)))
      EMAX2=1/(1+exp(-THETA(7)))

; transition 3 -> 2
X5032  = THETA(1) + ETA(1)
SL32   = THETA(2)
DF32   = (PKMETRIC/X5032)**SL32
D32    = EMAX1*DF32/(1+DF32)

; transition 2 -> 10
X50210 = THETA(3)
SL210  = THETA(4)
DF210  = (PKMETRIC/X50210)**SL210
D210   = EMAX2*DF210/(1+DF210)

; fraction of patients at 3 and 2 before treatment
P3B    = THETA(5)
P2B    = 1 - THETA(5)

FP33   = (1 - D32)
FP32   = D32 * (1 - D210)
FP310  = D32 * D210

FP23   = (1 - D32)
FP22   = (1 - D210 - (1 - D32))
FP210  = D210

FCHK1  = FP33+FP32+FP310+FP23+FP22+FP210-1 ; -1 compensates for 2 initial states

; fractions in respective bins
IF (DV.EQ.32)              Y=P3B *FP32    ; 3 -> 2
IF (DV.EQ.31.OR.DV.EQ.30)  Y=P3B *FP310   ; 3 -> <2
IF (DV.EQ.33)              Y=P3B *FP33    ; 3 -> 3
IF (DV.EQ.23)              Y=P2B *FP32    ; 2 -> 3
IF (DV.LT.22)              Y=P2B *FP210   ; 2 -> <2
IF (DV.EQ.22)              Y=P2B *FP22    ; 2 -> 2

FX3   = P3B*FP33 + P2B*FP23
FXN3  = 1-FX3

FX2   = P3B*FP32 + P2B*FP22
FXN2  = 1-FX2

FX10  = P3B*FP310 + P2B*FP210
FXN10 = 1-FX10

$THETA (0, 295, 1000)   ; X5032
       1 FIX            ; SL32
       (0, 1380, 10000) ; X50210
       1 FIX            ; SL210
       (0, 0.516, 1)    ; prob at 3
       <<REPLACE_DURING_LIKELIHOODPROFILING>> ; EMAX1
       <<REPLACE_DURING_LIKELIHOODPROFILING>> ; EMAX2

$OMEGA 0 FIX

$ESTIMATION MAX=9990 SIGDIG=3 METH=1 LIKE LAPLACE NUMERICAL NOABORT
$COVARIANCE
```

***tdmore* R code for the sensitivity analysis**

<please see separate files – PDSensitivity.zip>

***tdmore* R code for the simulations**

<please see separate files – simulation.zip>
